# Technologies for Joining and Forming Thin-Walled Structures in the Construction of Transportation Vehicles

**DOI:** 10.3390/ma16134594

**Published:** 2023-06-26

**Authors:** Dariusz Fydrych, Andrzej Kubit, Ján Slota, Agnieszka Kowalczyk

**Affiliations:** 1 Institute of Manufacturing and Materials Technology, Faculty of Mechanical Engineering and Ship Technology, Gdańsk University of Technology, 80-233 Gdańsk, Poland; 2 Department of Manufacturing and Production Engineering, Rzeszow University of Technology, 35-959 Rzeszów, Poland; 3 Institute of Technology and Materials Engineering, Technical University of Košice, 040 01 Košice, Slovakia; 4 Department of Chemical Organic Technology and Polymeric Materials, Faculty of Chemical Technology and Engineering, West Pomeranian University of Technology in Szczecin, 70-322 Szczecin, Poland

The pursuit of COx reduction has progressed the construction of transport systems produced using various types of materials to ensure weight reduction while maintaining sufficient functional and quality features [1,2]. When introducing new design solutions, economic issues are extremely important, requiring the use of cost-effective material solutions in mass-produced products. Consequently, there is currently a great demand for the development of technologies that enable the effective shaping and joining of often different construction materials in order to ensure the optimization of the structure in terms of weight, strength, stiffness, and manufacturing costs. 

Reducing the weight of the structure is especially important for vehicles, as it contributes to their speed, lifting capacity and operating costs. One of the methods achieving this is to use materials with a lower density or higher strength [3,4,5]. However, such a substitution may cause problems in the fabrication of the structure, in particular, limited weldability and formability issues [6,7,8].

Some light-metal alloys have limited weldability when using traditional arc-welding processes. In this case, it is particularly advantageous to use solid-state welding processes, e.g., variants of friction welding, in particular friction stir welding (FSW) [9,10,11]. In the last decade, FSW is being increasingly employed in the construction of, e.g., aircraft and automotive structures [12,13]. The growing interest in this method of joining is fully justified due to a number of advantages, such as the low energy consumption of the process and the possibility of joining different metals, plastics or composites [14,15]. In order to achieve the high quality of the joint, it is important to ensure appropriate welding conditions, from the geometry of the tool to the parameters of the welding process. The authors of work [14] carried out multi-criteria optimization of FSW welding parameters. When implementing a lap joint using EN AW-2024-T3 aluminum alloy sheets, they conducted experimental tests, including static and metallographic tests, for 27 different combinations of parameters. They considered the following parameters: pin length, welding speed and tool rotational speed. The aim of the optimization was to discover a combination that ensured high joint strength with the minimal dispersion of test results, and quality in terms of the joint microstructure.

In paper [16], the authors analyzed the effect of the tool shape and process parameters on the mechanical properties of AW-3004 aluminum alloy. This was the first time that this material had been joined using a FSW method. It was discovered that joints made with a cylindrical threaded tool pin had a higher tensile strength and elongation than those made with a tapered threaded pin. Moreover, it was proved that rotational speed may be the key parameter in improving welding conditions and the mechanical properties of aluminum alloy joints.

One of the methods of reducing the weight of the structure is to perform dissimilar welded joints [17]. The subject of work [18] was the numerical analysis of thermal history of AA1100 aluminum alloy and St-14 low-alloy steel FSW joints. It was found that the mechanical work on the joint line increased with the pin angle and larger stir zone forms. Additionally, it was shown that the maximum heat was generated on the steel side.

A variation of FSW linear welding is refill friction stir spot welding (RFSSW). Kubit et al. [19] presented studies on the possibility of replacing riveted joints using RFSSW technology in the construction of a semi-monocoque aircraft fuselage in a skin-stringer joint. For selected parameters of the RFSSW process, the row joint of stringers with a skin made of aluminum alloy EN AW-7075-T6 Alclad was employed. The stiffened plate was subjected to static tests in the uniaxial compression test. The plates were compared with welded and riveted stringers. Experimental studies using 3D digital image correlation deformation analysis demonstrated that RFSSW joints can be an effective alternative to riveted joints. At the same time, they confer two main benefits: they eliminate rivet holes, which are stress concentrators, and they also enable the weight of the structure to be reduced. In the same work, FEM numerical analyses were performed to determine the minimum spacing between spots at which inter-spot buckling does not occur.

The need to minimize the mass of vehicles applies not only to civil transport, but also to military transport. A lower weight facilitates logistics activities and also leads to a reduction in energy consumption and, finally, can lead to increased transport capacity. The authors of paper [20] presented the results of research investigating the ballistic efficiency of plate structures composed of layers with different properties, conducted in the search for alternative solutions to armor steel, which has at least twice the density of the investigated materials. The paper investigated the extent to which the considered layered coatings absorbed the energy of a 7.62 mm bullet. Holes were also analyzed to determine the mechanism of energy absorption. Finally, numerical FEM analyses were carried out, which enabled a more comprehensive understanding of the physical phenomena occurring at the subsequent stages of projectile energy absorption.

In order to be industrial applicable to the field of transport, the development of economically justified plastic shaping and joining technologies is required for each group of materials, with the ability to combine these with other materials. One promising group of materials are metal-plastic composites (MPCs), with significant advantages such as a relatively low weight compared to steel, and mechanical properties comparable to structural aluminum alloys [21]. The primary MPC composite is LITECOR^®^ developed by ThyssenKrupp Steel Europe for applications in the automotive industry. This MPC consists of two layers of HX220YD interstitial steel sheets (0.2–0.5 mm) with a polyamide (PA)/polyethylene (PE) intermediate layer (52 wt.% PA6, 36 wt.% PE and 12 wt.% other additives) [21]. Currently, such materials are joined by mechanical fastening (e.g., self-piercing riveting, clinching), joining by forming (e.g., hemming, seaming), and adhesive bonding [21,22,23,24].

Kustroń et al. [21] extensively researched the possibility of welding LITECOR^®^ composites with steel using resistance spot welding (RSW) technology. However, due to their polymer core, it is not possible to weld directly with this method. Therefore, hybrid welding was performed with the use of an ultrasonic head, causing local melting of the polymer layer in the first stage of joining, thanks to which it was possible to obtain metal contact between the linings. Subsequently, welding was carried out using the RSW method. The discussed paper presents the influence of various parameters on the properties of welded joints of the LITECOR^®^ composite with a sheet made of DP600 steel, demonstrating the effectiveness of the applied method of joining.

In addition to the technology of joining, it is extremely important to master the technology of shaping new materials. The authors of the work [25] conducted a series of tests to determine the tribological properties of the LITECOR^®^ composite under consideration. The aim of these studies was to determine the value of the friction coefficient under various friction conditions. Different friction surfaces, lubrication conditions, and clamping forces were considered. The results of the described tribological tests are intended to be used to determine the optimal parameters of plastic shaping for MPC composites.

In turn, Kubit et al. [26] described the results of experimental research in which they produced stiffening ribs in the LITECOR^®^ composite using the incremental sheet forming (ISF) method. They considered various parameters of the shaping process and analyzed their influence on the distribution of surface residual stress across the ribs and on the properties of the surface. As part of the analysis, they proposed the effective parameters for the formation of stiffening ribs, while demonstrating the effectiveness of ISF technology in producing MPC composites.

Another aspect of the transport industry is the production of railway tracks. Tuz et al. described studies on the behavior of high-carbon rail steel during cutting processes [27]. The thermal cycle of oxyfuel cutting caused significant changes in the morphology and properties of the material. Unfavorable structures with high hardness reaching over 800 HV10 were found. The brittle structure combined with high stresses caused the formation of transcrystalline microcracks. The authors proposed the minimization of these unfavorable phenomena by extending the cooling time through preheating.

The issue of weldability in high-strength steels was the crux of two papers by an international team from Czech Republic and Slovakia [28,29]. The authors determined the structure–properties relationships for steel-grade S960MC HSLA subjected to simulated thermal cycles under welding conditions. The paper [28] presents experimental studies on the growth kinetics of the austenitic grain of S960MC steel in which the activation energy values required for grain growth Q and the proportional constant K0 were determined. In the second article [29], research results regarding the determination of the continuous cooling transformation diagram for the same steel grade were presented.

The conceptual study and manufacture of a configurable and weld-free-lattice base for automatic food machines was presented by Pirondi et al. [30]. The article describes the complete design process of the device: from its concept to its implementation and the verification of its operation for five years in industrial conditions.

In article [31], the dry sliding wear and friction behavior of Al7068/Si3N4/BN hybrid composites were studied, and the presented indicate that the developed hybrid composite could be a potential candidate material for various tribological applications, especially in the aerospace and automotive sectors.

The results presented in article [32] are especially valuable for the aviation and aerospace industry, because the elements of flying vehicles require high strength and corrosion resistance at the same time, which ensures the use of layers on high-strength materials. The subject of the work was the experimental research and numerical modeling of aluminum alloys with technically pure aluminum cladding during the bending test [32]. The study concluded that the models describing the phenomena during bending are more accurate than any to date.

The last two articles published in SI concern a modern and environmentally friendly method of obtaining adhesive binders for pressure-sensitive adhesives [33,34]. It has been proven that the light-induced telomerization and subsequent photocrosslinking of acrylate monomers is a particularly convenient method of obtaining thin adhesive films, with further potential applications in the automotive industry. It was found that an increase in the acrylic acid content causes significant changes in the reaction rate, monomer transformation, and the solid content in prepolymers and their viscosity, as well as in adhesion and cohesion [33]. In turn, in the second paper, the key factors determining the properties of solvent-free acrylic pressure-sensitive adhesives were indicated [34].

Papers published in this Special Issue have been cited 105 times so far, which demonstrates the importance of the topics presented. The continuation of the subject of this Special Issue is the MDPI Topic entitled: “Manufacturing and Processing of Materials for Transport Industry”, with the potential of article publication in the MDPI journals *Lubricants*, *Materials*, *Metals*, *Processes* or *Sustainability*. The quality of the articles is supervised by the international topic editorial board, in addition to the MDPI editorial office. 

We, the Special Issue editors, would like to thank all the co-authors of published papers, reviewers and academic editors. Additionally, we would like to thank the MDPI editorial staff for their invaluable support and commitment in the publishing process.
